# A pilot, randomized controlled trial of telementorship: A useful tool during social distancing

**Published:** 2021-01-20

**Authors:** Nicolas D. Prionas, Tiffany H. Kung, Ann Dohn, Nancy Piro, Rie von Eyben, Laurence Katznelson, Thomas J. Caruso

**Affiliations:** ^1^Department of Radiation Oncology, University of California San Francisco Medical Center 505 Parnassus Ave. San Francisco, CA; ^2^Department of Anesthesiology, Perioperative, and Pain Medicine, Massachusetts General Hospital, 55 Fruit Street, Boston, MA; ^3^Department of Graduate Medical Education, Stanford University Medical Center, 300 Pasteur Drive - Room HC435, Stanford, CA; ^4^Department of Radiation Oncology, Stanford Cancer Center, MC 5847, 875 Blake Wilbur Drive Stanford, CA; ^5^Department of Medicine, Stanford University Medical Center, 300 Pasteur Drive - Room S102, Stanford, CA; ^6^Department of Neurosurgery, Stanford Neuroscience Health Center, 213 Quarry Road, Palo Alto CA; ^7^Department of Anesthesiology, Perioperative, and Pain Medicine, Stanford University Medical Center, 300 Pasteur Drive, H3584 Stanford, CA

**Keywords:** Mentorship, education, telehealth, social distancing

## Abstract

**Background:**

During social distancing, resident mentorship may be an unmet need. Telementorship, mentorship through video conferencing software, presents a unique approach to overcome these challenges.

**Aims:**

This study evaluated whether telementorship through video conference increased access to mentorship encounters and decreased perceived barriers to access, factors that determine likelihood to maintain mentor relationships, and quality of mentorship.

**Methods:**

A year-long randomized, prospective cohort study was conducted in 2016–2017 with pairs of resident mentors from seven different training programs and medical student mentees, randomized to telementorship or in-person mentorship. The number of quarterly encounters was monitored and demographic predictors of meeting were determined. Likert scale survey responses were analyzed with linear regression.

**Results:**

Forty-three of 46 (93.5%) volunteer mentor-mentee pairs participated. Telementorship did not alter likelihood of meeting or attitudes toward mentorship barriers (time and distance). Mentee satisfaction increased from 42.5% to 65.4% (*P*<0.05) throughout the year. Operating room-based practice (*P*<0.05) and higher postgraduate level (*P*=0.02) decreased the likelihood of meeting.

**Conclusion:**

Telementorship provided an equal number of encounters compared to the pairs who were asked to meet in-person. Telementorship may serve as an adjunct modality for flexible communication.

**Relevance for Patients:**

Medical mentorship is a key component to medical education. Effective mentorship increases academic research productivity, job satisfaction, and advancement of clinical skills, which translate to improved patient care.

## 1. Introduction

Mentor relationships are mutually beneficial to mentees and mentors [1]. In medical school, mentees experience professional development, psychosocial support, increased interest in research, and career counseling [2-4]. Mentor benefits include improvement in leadership skills and enhanced academic productivity, which develops professionalism, increases students’ interest in research, and supports personal growth [5-7]. Frequently cited barriers to mentorship include time limitations and physical distance, which are exacerbated by medical school and residency training schedules, and likely worsened by the recent social distancing guidelines [8-11].

Telementorship, which is mentorship through video conference, presents a unique approach to reduce these barriers. The majority of mentorship literature in medicine focuses on training faculty to become better mentors [12-14]. However, there is little literature that evaluates telementorship for professional mentorship of medical students. When trainees are studied, they are commonly in the mentee role within specific training programs [15-18]. Incorporation of a formal mentorship curriculum into residency training is challenging due to the heterogeneity of residency programs. Telementorship may alleviate some of the scheduling constraints of in-person mentorship that is exacerbated by busy trainee and medical student curricula.

We randomized resident and medical student mentorship pairs to in-person and video conference. Our aims were to determine if access to telementorship through video conference increased the number of mentorship encounters and decreased perceived barriers to access, identify factors that determine likelihood to maintain mentor relationships, and quantify the quality of mentorship.

## 2. Methods

### 2.1. Context

We conducted a prospective, randomized cohort study at our academic institution between September 2016 and June 2017, which has over 1300 medical trainees in 111 Accreditation Council for Graduate Medical Education (ACGME) accredited programs. The GME deployed a mentorship curriculum to 46 volunteer residents from seven GME training programs. Forty-six pre-clinical medical students volunteered as mentees. The Institutional Review Board granted a waiver of approval for this study.

### 2.2. Mentorship curriculum

Curriculum components included online learning, in-person interactive seminar, online just-in-time (JIT) modules, and experiential learning. Resident mentors initially completed an online module distributed by the University of Minnesota [19]. The module uses text, audio, and interactive activities to engage learners in understanding mentoring models and strategies to address common mentorship challenges. Resident mentors and medical student mentees were also invited to an hour-long, in-person interactive seminar which discussed techniques for successful mentorship relationships [20-22]. Resident mentors and medical student mentees met quarterly. Before each mentorship meeting, residents completed JIT learning modules consisting of readings from a mentorship review article with comprehension questions [23]. JIT module topics included advocacy, role modeling, race/ethnicity in mentorship, and the natural course of a mentorship relationship.

### 2.3. Establishment of mentoring relationships

Mentor-mentee pairing was designed to be organic and mentee driven [14]. The initial in-person seminar was followed by a networking event that encouraged resident and medical student mingling to identify compatible pairings. Before the seminar, medical students were also provided with a list of resident mentor profiles, which included educational background, department, research interests, and hobbies. Medical students identified a list of desired mentors and pairings were made according to medical student request. In the absence of a mentor request from a medical student, they were paired according to concordant professional interests and hobbies.

### 2.4. Mentorship encounters

Pairs were randomized by random number generator using Excel 2016 (Microsoft Corporation, Redmond, WA, USA) to in-person or telementorship quarterly meetings. No restrictions were placed on the video conferencing software used for telementorship. The content discussed at each meeting was guided by a discussion form emphasizing core foci of mentorship including clinical knowledge, research opportunities, career planning, networking/exposure, sponsorship, and wellness [19-22].

### 2.5. Measures

#### 2.5.1. Demographics

Gender, race, year of training in residency or medical school, presence of pre-existing mentor relationships, and type of resident training program were collected as participant characteristics.

#### 2.5.2. Aim 1: Telementorship versus In-person mentor encounters

The effectiveness of telementorship to overcome traditional time and distance barriers was assessed by two methods. First, we compared the number of mentorship encounters between the in-person and telementorship groups. Second, we surveyed participant attitudes toward barriers, at 3 time points (Table 1).

**Table 1:** Survey questions assessing mentorship barriers

	Not at all	Barely	A little	Some what	Very	Extremely
Time commitment						
Physical distance						
Lack of skills/training						
Gender challenges						
Race/ethnicity						
challenges						

#### 2.5.3. Aim 2: Determinants of likelihood to maintain mentor relationships

Postgraduate year (PGY) of training, practice setting (operating room based or clinic based), gender, race, and type of undergraduate and medical school training (public or private) were examined as predictors of mentorship meeting.

#### 2.5.4. Aim 3: Quality of mentorship

We measured overall mentoring confidence as reported by the resident mentor and as perceived by their medical student mentee. Second, we measured mentee confidence in the six core areas of mentorship at the 3 time points throughout the year.

### 2.6. Analysis

All surveys used a 6-point, 1-6 Likert scale that ranged from “not at all [1]” to “extremely [6]” in regard to frequency or effectiveness. Responses were binned into three groups with those receiving a 5 or 6 denoting a high degree of effectiveness. Generalized linear regression models accounting for repeated measures were performed. Power analysis suggested 17 subjects needed to detect a 0.5 increase in continuous Likert score with 90% power. Intention to treat analysis was used. All statistics were performed in SAS version 9.4 (Cary, NC, USA). Study data were collected and managed using REDCap electronic data capture tools [24].

## 3. Results

### 3.1. Demographics

Three pairs of residents and medical students withdrew from the study after randomization (2 in-person group and 1 telementorship group), citing time limitations, or lack of desire to participate, for a total of 43 of 46 participating pairs (93.5%). Residents participated from seven ACGME programs, including anesthesiology (11, 24.4%), ophthalmology (4, 8.9%), pediatrics (15, 33.3%), physical medicine and rehabilitation (1, 2.2%), plastic surgery (3, 6.7%), psychiatry (4, 8.9%), and radiation oncology (7, 15.6%) (Table 2).

**Table 2:** Participant demographics. †PGY for residents, school level for medical students

	Residents; *n* (%)	Medical students; *n* (%)
Female	27 (67.5)	27 (65.9)
Race		
Caucasian	17 (42.5)	12 (29.3)
African American	2 (5.0)	1 (2.4)
Asian/Pacific Islander	16 (40.0)	19 (46.3)
Latino/Hispanic	3 (7.5)	3 (7.3)
Native American	0 (0)	1 (2.4)
Other	2 (5.0)	4 (9.8)
Declined	0 (0)	1 (2.4)
Year†		
1	13 (28.9)	3 (7.3)
2	14 (31.1)	35 (85.4)
3	9 (20.0)	3 (7.3)
4	7 (15.6)	0 (0)
5	1 (2.2)	
6	1 (2.2)	
Had a mentor before study	31 (77.5)	36 (87.8)

### 3.2. Telementorship versus in-person mentor encounters

**Figure 1:**
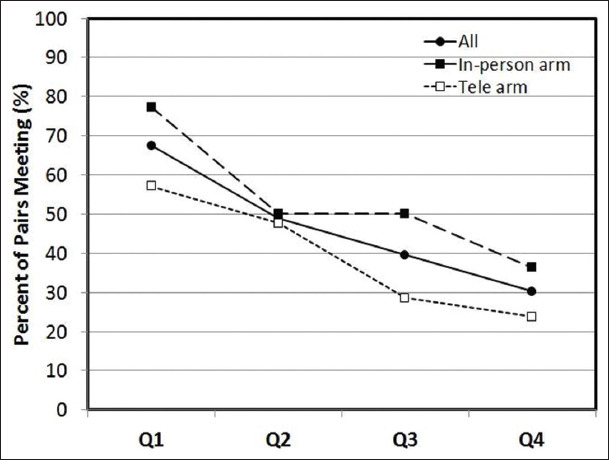
Proportion of mentor-mentee pairs meeting each quarter in the in-person group (closed square), telementorship group (open square), and in all participants (circles). Mentorship encounters were similar for both types of encounters.

There was no difference in number of mentorship encounters between the telementorship and in-person groups (*P*=0.35) (Figure 1).

Residents and medical students reported time commitment and distance as the most significant barriers to mentorship (Table 3).

**Table 3:** Proportion of residents and medical students reporting specific barriers to mentorship as very or extremely significant over time

	Resident				Medical student			
	
	Baseline; *n* (%)	Mid-program; *n* (%)	End; *n* (%)	*P*	Baseline; *n* (%)	Mid-program; *n* (%)	End; *n* (%)	*P*
Barriers to mentorship:								
Time commitment	21 (52.5)	11 (50.0)	12 (57.1)	0.94	22 (55.0)	14 (45.2)	13 (46.4)	0.94
Distance	11 (27.5)	8 (36.4)	7 (33.3)	0.58	11 (27.5)	7 (22.6)	10 (35.7)	0.49
Lacking training	10 (25.0)	5 (22.7)	2 (9.5)	0.25	16 (40.0)	10 (32.2)	7 (25.0)	0.35
Gender challenges	1 (2.5)	1 (4.5)	1 (4.8)	0.91	3 (7.5)	2 (6.5)	2 (7.1)	1.00
Race challenges	0 (0)	1 (4.5)	1 (4.8)	NS	3 (7.5)	5 (16.1)	3 10.7)	0.45

The proportion of mentorship pairs meeting quarterly decreased from 67.4% in quarter 1 to 30.2% in quarter 4 (*P*<0.001, Figure 1). There was no significant change in attitudes toward perceived barriers overtime**.**

### 3.3. Determinants of likelihood to maintain mentor relationships

Residents working in operating room-based practices (*P*<0.05) and of higher PGY level (*P*=0.02) were less likely to meet with their mentee overall, while other factors were not significant predictors of meeting.

### 3.4. Quality of mentorship

**Figure 2:**
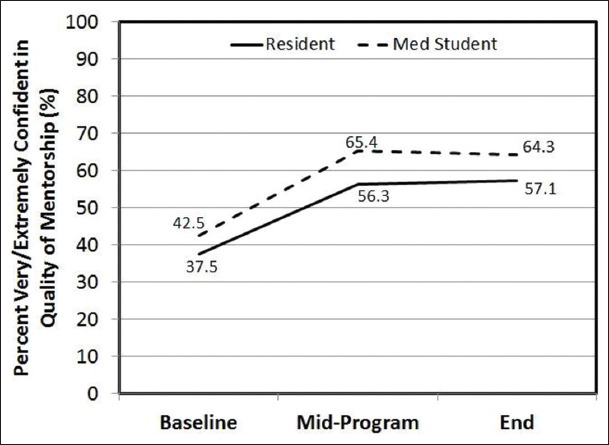
Resident and medical student-reported confidence and satisfaction, respectively, in the mentorship delivered were similar (*P*=0.99) with a significant improvement in medical student scores overtime (*P*<0.05) and improvement among resident self-reports overtime (*P*=0.05).

The percentage of residents who felt very or extremely confident in their mentorship skills increased from 37.5% at baseline to 56.3% (*P*=0.05) by the midpoint of the program and remained unchanged at curriculum completion. Overall, mentee satisfaction of their resident mentor increased significantly from 42.5% at baseline to 65.4% (*P*<0.05) by the midpoint and remained unchanged at curriculum completion (Figure 2).

**Figure 3:**
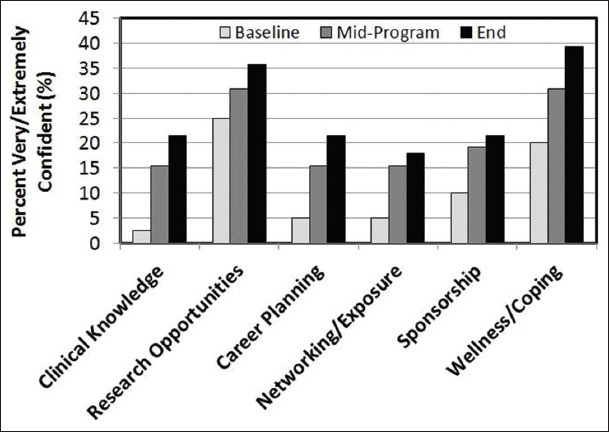
Mentee-reported confidence in core professional domains increased significantly overtime for clinical knowledge, career planning, networking/exposure, sponsorship, and wellness/coping.

Medical students reported improved confidence in all professional domains regardless of group assignment (Figure 3). Confidence increased significantly overtime for clinical knowledge (*P*<0.01), career planning (*P*<0.001), networking/exposure (*P*=0.01), sponsorship (*P*<0.001), and wellness/coping (*P*=0.02).

## 4. Discussion

This study evaluated telementorship for professional mentorship of medical students, in contrast to most studies of telementoring in medicine that evaluates the effectiveness of remotely training a particular skill or surgical technique [25-27]. The substitution of telementorship for in-person encounters did not alter the likelihood of meetings, indicating that mentorship was just as likely to occur remotely as in-person. Even though the type of meetings that occurred between each group was not significantly different over the year, the second quarter meeting frequency was nearly identical in occurrence. We attribute this to chance as there were no other external factors that influenced the participants more so during the second quarter. In addition, because the differences in meetings each quarter were statistically insignificant, we believe that the difference in meetings each quarter was due to chance. Regardless of group assignment, medical student mentees had increased confidence in each core domain that was examined (Figure 3).

Commonly published barriers to mentorship, including the time cost of arranging in-person mentorship encounters and distance, were confirmed as barriers in this study [8-10]. The number of mentorship encounters did reduce throughout the year. We hypothesize that given 85% of medical student mentees who were second year medical students, their academic focus shifted to studying for the USMLE step two examination. Increased PGY level and OR-based specialties were less likely to maintain quarterly meetings, which may be attributed to less scheduling flexibility. Interestingly, more female resident mentors *n*=27 (67.5%) and female medical student mentees *n*=27 (65.9%) participated in the study than males, which has not been well documented in the literature. Further studies are needed to clarify if this was a local phenomenon or representative of a larger trend.

As medical trainees face tremendous physical and psychological pressure in the COVID-19 pandemic, limited social contact can further strain mental health and well-being [28]. Telementorship may serve as an adjunct modality for flexible communication, which can be particularly helpful during times of social distancing. A self-deprecating trend was observed in resident-reported confidence in their mentorship skills, with scores consistently lower than corresponding medical student-reported satisfaction with the mentoring received (Figure 2). It is unknown if this is a local phenomenon or a symptom of a wider endemic of resident well-being. The majority of residents and medical students were interested in continuing their mentor relationship, encouraging the development of an ongoing culture of mentorship training for residents. With broader deployment of the curriculum across GME, mentorship may shift from an “accidental leadership” skill to a core competency developed during medical training [29].

This study had several limitations. First, the sample size was small and there was attrition. As anticipated, there was a decline in mentorship meetings overtime. However, participation rates in previously published mentorship studies have reported similar attrition [1,30]. Second, as a pilot study, there were uninvestigated areas, including the length of the mentorship meetings between groups and additional meetings outside of the assigned quarterly meetings. Given the volunteered time of the mentors and mentees and number of other surveys being administered, we limited the number of outcomes measured in this pilot study. It is possible that unmeasured data, including meetings outside of the formal program, may have biased the results in unpredictable directions. Third, this study recruited volunteers, which may have resulted in selection bias, favoring participants who already value mentorship. Fourth, the survey instruments were not tested for construct validity before utilization in this study. All attempts were made to utilize clear language with scale anchors. There were no queries or clarifications by participants. Fifth, this study enabled mentors and mentees to meet face to face and engage in activities before transitioning to a virtual relationship, which distinguishes our approach from fully online mentorship. Last, the intervention was performed at a single institution. The results may not be transferrable to other institutions with different demographics.

## 5. Conclusion

We compared an in-person and telementorship program composed of residents of various departments and training years and medical students. The results indicate that the frequency of telementorship encounters did not differ from in-person meetings, nor was there a change in perceptions of mentorship barriers overtime. Telementorship may serve as an adjunct modality for flexible communication during periods of social restriction.
